# A multi-biomarker approach to risk stratification and detection of early cardiac disease in systemic sclerosis

**DOI:** 10.1371/journal.pone.0328734

**Published:** 2025-07-31

**Authors:** Justin K. Lui, Fatima El-Adili, Matthew Cozzolino, Morgan Winburn, Marcin A. Trojanowski, Deepa M. Gopal, Michael P. LaValley, Elizabeth S. Klings, Andreea M. Bujor

**Affiliations:** 1 Pulmonary Center, Boston University Chobanian and Avedisian School of Medicine, Boston, Massachusetts, United States of America; 2 Arthritis and Autoimmune Diseases Center, Boston University Chobanian and Avedisian School of Medicine, Boston, Massachusetts, United States of America; 3 Section of Rheumatology, Boston University Chobanian and Avedisian School of Medicine, Boston, Massachusetts, United States of America; 4 Section of Cardiovascular Medicine, Boston University Chobanian and Avedisian School of Medicine, Boston, Massachusetts, United States of America; 5 Department of Biostatistics, Boston University School of Public Health, Boston, Massachusetts, United States of America; Nippon Medical School, JAPAN

## Abstract

**Objective:**

We sought to investigate the relationship between serum biomarkers of cardiac dysfunction, longitudinal strain on echocardiography, and all-cause mortality in patients with systemic sclerosis.

**Methods:**

This was an observational study using a biorepository of serum samples of patients with systemic sclerosis who underwent echocardiography. We investigated 3 biomarkers: periostin, galectin-3, and N-terminal prohormone brain natriuretic peptide and applied a K-means clustering resulting in 3 patient clusters. We subsequently measured left ventricular and right ventricular free wall longitudinal strain in each cluster. We then determined the association between each cluster and time to all-cause mortality compared to N-terminal prohormone brain natriuretic peptide, alone.

**Results:**

The 125 patients with systemic sclerosis included in the study were divided into 3 clusters based on biomarker levels (Cluster 1: N = 75; Cluster 2: N = 39; Cluster 3: N = 11). Compared to Cluster 1, Cluster 2 had only elevated periostin levels whereas Cluster 3 had elevated levels of all 3 serum biomarkers and was characterized by reduced left ventricular and right ventricular free wall longitudinal strain, regionally and globally. When adjusted for age, sex, systemic sclerosis disease duration, and forced vital capacity, patients in Cluster 3 had a HR of 14.42 (95% CI: 4.82, 43.18) for all-cause mortality compared to those in Cluster 1.

**Conclusion:**

In conclusion, combining N-terminal prohormone brain natriuretic peptide, periostin, and galectin-3 as serum biomarkers enhances risk stratification and sensitivity in detection of cardiac disease in patients with systemic sclerosis. However, before implementation in routine care, further prospective studies must refine biomarker sensitivity, specificity, and accuracy together with optimizing detection strategies and establishing clinical protocols for integration.

## Introduction

Systemic sclerosis (SSc) is a heterogeneous, multiorgan connective tissue disease. Pulmonary hypertension (PH) is one of the leading causes of mortality in this patient population that may be worsened by concurrent cardiac dysfunction [1–3]. However, cardiac dysfunction, specifically left ventricular (LV) dysfunction, is often complex in this patient population [4]. LV dysfunction can occur from primary SSc cardiac involvement, which may result in elevated left atrial pressures, pulmonary venous hypertension, and subsequent right ventricular (RV) dysfunction [5]. The resultant RV pressure overload may transform the LV geometry by creating stress along the interventricular septum, giving rise to reduced LV contractility and impaired filling [2,6]. While pulmonary vasodilator therapy may effectively reduce RV pressures by increasing pulmonary blood flow, it can, in turn, lead to increased left-sided filling pressures, thereby exacerbating LV dysfunction in a potentially self-perpetuating cycle with consequent pulmonary edema [5]. Therefore, identifying concurrent primary SSc cardiac involvement is critical, particularly among those affected by PH.

Primary SSc cardiac involvement clinically affects 20–25% of patients with SSc [7,8]. However, greater than 70–80% of patients with SSc have histopathologic myocardial fibrotic changes at autopsy, suggesting that much of the disease is subclinical [9,10]. Endomyocardial biopsy, an invasive procedure with inherent risks, remains rarely used for diagnosing cardiac disease [11–13]. Additionally, the capability of conventional echocardiography in detecting subclinical cardiac disease is limited. Advanced imaging approaches including cardiac magnetic resonance imaging [11,14–16] and strain imaging using speckle tracking echocardiography [2,16] may be more effective at capturing subclinical disease, but they continue to be underutilized. Hence, there is a need to identify and characterize cardiac biomarkers to complement current imaging modalities to more effectively distinguish and monitor for early cardiac disease in SSc.

Brain natriuretic peptide and its precursor N-terminal prohormone brain natriuretic peptide (NT-proBNP) continue to be two main cardiac biomarkers used in risk stratifying patients with cardiopulmonary disease. However, the challenge is often in differentiating between primary SSc cardiac involvement and PH, both of which are associated with increased mortality risk [17]. Furthermore, NT-proBNP can be abnormal in various other non-cardiopulmonary conditions as well including, for example, cerebrovascular disease, renal insufficiency, and cirrhosis [18]. Finally, NT-proBNP may be elevated much later into the disease course, typically released by cardiac myocytes in response to stretching of the myocardial wall as a consequence of elevated left atrial pressures [19]. To date, there have not been any studies in using NT-proBNP for identifying early cardiac disease that can be captured by cardiac strain abnormalities.

Hence, there has been significant interest in identifying circulating proteins, particularly those representing early fibrotic changes, to advance our understanding of SSc. A growing body of evidence has been highlighting the potential of periostin [20,21] and galectin-3 [22–25] as biomarkers for fibrosis associated with SSc primary cardiac involvement. In an autopsy study investigating sudden cardiac death, two proteins, periostin and galectin-3, were highly expressed in fibrotic cardiac tissue [26]. In patients with SSc, we found elevated periostin expression in cardiac tissue and a strong correlation between serum periostin levels and LV mass index [20]. Circulating galectin-3 has emerged as a valuable marker for risk stratification and prognostic evaluation of patients with congestive heart failure unrelated to SSc [27]. To date, no studies have investigated the expression of galectin-3 in SSc cardiac tissue, but elevated circulating levels were reported to correlate with diastolic dysfunction in SSc [27]. The objectives of this study were to: 1) Determine whether incorporating galectin-3 and periostin with NT-proBNP improves classification of cardiac dysfunction by cardiac strain compared to NT-proBNP, alone; and 2) Compare mortality risk stratification of patients with SSc by a multi-biomarker approach using periostin, galectin-3, and NT-proBNP versus NT-proBNP, alone.

## Patients and methods

This was a retrospective, observational single-center study using patients who were enrolled into the Scleroderma Center of Research Translation clinical database at Boston Medical Center/Boston University Chobanian & Avedisian School of Medicine. The study was approved by the Institutional Review Board (H-31479) at the Boston University Medical Campus and Boston Medical Center. Patients were consented at the time of database enrollment. Access to information that could identify individual participants were available during or after data collection. Identifiable data used for this study was accessible between April 7, 2020 and April 5, 2025. All patients were diagnosed with SSc according to the American College of Rheumatology/European League against Rheumatism collaborative criteria [28]. These patients were subsequently included in the current study if they had both an available serum biospecimen (between the dates of August 31, 2012 and December 31, 2018) and an echocardiogram performed at Boston Medical Center. These patients were followed until the end date of January 1, 2023. Patients who received a heart and/or lung transplant were excluded from the study.

### Biomarker protein assays

Serum samples were collected from patients with SSc enrolled in the Scleroderma Center of Research Translation database. Blood was collected using standard venipuncture techniques and was allowed to clot at room temperature for 30 minutes before being centrifuged at 1500 x g for 10 minutes to separate the serum. The resulting serum was aliquoted into prelabeled cryovials to ensure proper identification and traceability. Each aliquot was immediately frozen and stored at −80°C to preserve the integrity of the proteins. Upon thawing, each serum biospecimen was measured for NT-proBNP (EHPRONPPB, Thermo Fisher Scientific, Waltham, MA), periostin (EHPOSTN, Thermo Fisher Scientific, Waltham, MA), and galectin-3 (BMS279-4, Thermo Fisher Scientific, Waltham, MA) using enzyme-linked immunosorbent assays at 1:100 dilution in duplicates in accordance with the manufacturer’s protocol for each of the respective assays. Absorbance was measured on a microplate reader (Multiskan EX, Thermo Scientific, Vantaa, Finland).

### Clinical data, definition, and outcomes

All-cause mortality from the time of serum biospecimen collection was the primary outcome of this study. Within our entire patient cohort, we collected the following data: 1) Demographics; 2) Overlapping autoimmune conditions; 3) Comorbid conditions; 4) Antibody positivity; 5) Pulmonary function testing; and 6) Functional capacity. We obtained echocardiograms closest to the time of biospecimen collection and compiled data on LV and RV free wall longitudinal strain in addition to conventional parameters. Additionally, we also recorded measures of cardiopulmonary hemodynamics obtained on right heart catheterization, when available. Briefly, diffuse SSc was defined by the LeRoy classification [29] and SSc disease duration was determined from the initial non-Raynaud’s symptoms (or from the time of SSc diagnosis, if unavailable) to the time of biospecimen collection. SSc-PH was defined by a mean pulmonary artery pressure > 20 mmHg on right heart catheterization. SSc-related interstitial lung disease (SSc-ILD) was identified based on diagnosis by a rheumatologist and/or pulmonologist. All other comorbid conditions were identified based on prior documentation by any physician within the patient’s care team.

For each echocardiogram, we computed LV and RV free wall longitudinal strain through Philips QLAB, an embedded software plug-in within Philips Xcelera (Amsterdam, Netherlands) that integrates with TOMTEC-ARENA TTA2.42 (Unterschleissheim, Germany). After assigning an automated electrocardiographic gating to time events in the cardiac cycle, the software employs an automated edge detection algorithm for the segmentation of the endocardial border. LV longitudinal strain was obtained over a 16-segment model using 3 views: 1) Apical-2-chamber; 2) Apical-3-chamber; and 3) Apical-4-chamber. RV free wall longitudinal strain was obtained over a 3-segment model using a RV-focused apical-4-chamber view or standard apical-4-chamber view (when the RV-focused apical-4-chamber view was unavailable or suboptimal). We inspected all images for any errors in segmentation and provided manual corrections for optimization of border tracking. These data were obtained in accordance to the American Society of Echocardiography guidelines [30]. Abnormal LV function was defined by a LV GLS < 18% [2], and abnormal RV function was defined by a RV free wall GLS < 20% [31]. Lastly, we determined LV diastolic dysfunction based on the LV ejection fraction (normal defined as ≥ 50%), left atrial (LA) volume index, and tissue Doppler, as previously described [32] LA dilation was defined by a LA volume index threshold > 34 mL/m^2^ [30]. Valvular disease (mitral, tricuspid, pulmonic, and aortic) was present if it was at least mild in severity.

### Statistical analysis

For continuous variables, we used mean (± standard deviation) to summarize normally distributed data and median (interquartile range [IQR]) for non-normally distributed data. A one-way analysis of variance (ANOVA) was used to compare normally distributed data, and a Wilcoxon test was used to compare non-normally distributed data. For categorical variables, we summarized the data using frequency and percentages calculated from the number in each cluster. A chi-squared test was used to compare groups. When the sample size was small, a Fisher’s exact test was applied instead. In the event of missing data, we used multiple imputation by chained equations approach (See S1 Table for summary of missing data). For variables with >15% missing, we also applied a logistic regression to determine whether the variable is missing completely at random, using the following basic demographics: a) Age; b) Male sex; c) Diffuse SSc; d) SSc disease duration; and e) PH.

For the clustering of biomarkers, we first performed a logarithmic transformation given the difference in magnitude and skewness of the data (See S1 Fig). We then applied a K-means clustering. The number of clusters was first determined by the elbow method and confirmed using the Calinski-Harabasz index (See S2 Fig) [33,34]. We used principal component analysis to visualize our resultant clustering (See S3 Fig). As a comparator to our multi-biomarker clusters, we also divided patients with SSc based on NT-proBNP, alone, into low (< 300 pg/mL), intermediate (≥ 300 pg/mL and < 1,100 pg/mL), and high (≥ 1,100 pg/mL) risk NT-proBNP groups. Between each cluster and between each NT-proBNP risk group, we performed a one-way ANOVA test and a post hoc Tukey’s test to delineate differences in LV and RV free wall longitudinal strain. To compare the multi-biomarker clustering with using isolated NT-proBNP risk stratification, we calculated the sensitivity, specificity, and accuracy in detecting abnormal LV and RV function by LV and RV free wall GLS, respectively.

We initially applied the Kaplan-Meier method for constructing survival curves for each cluster to determine associations with all-cause mortality, the primary endpoint of this study. Patients who survived were censored at the end of the study (January 1, 2023) or at the date of their last visit prior to being lost to follow-up. We tested statistical significance using a log-rank test. We then used a Cox proportional hazard regression model to determine the hazard ratio (HR) for all-cause mortality. The model was adjusted for the following covariables: 1) Age; 2) Male sex; 3) SSc disease duration; and 4) Forced vital capacity (FVC). Specifically, FVC was used as a surrogate for ILD severity which may be a confounder given that both SSc-ILD and SSc-PH are major contributors to mortality in this patient population. Each variable including cluster number was evaluated for proportional hazards assumption by Schoenfeld residuals. We also performed two sensitivity analyses: the first to exclude patients who were lost to follow-up; and the second to exclude patients who had greater than a 1-year time window between echocardiography and serum collection. Statistical significance was determined by a *p*-value < 0.05. R version 4.3.0 (R Core Team, Vienna, Austria) was used for all analyses. The ‘mice’ package was implemented for multiple imputation employing 5 iterations from which a mean value was calculated. Survival analyses were performed using the ‘epiR’, ‘survival’, and ‘survminer’ packages.

## Results

**Fig 1:**
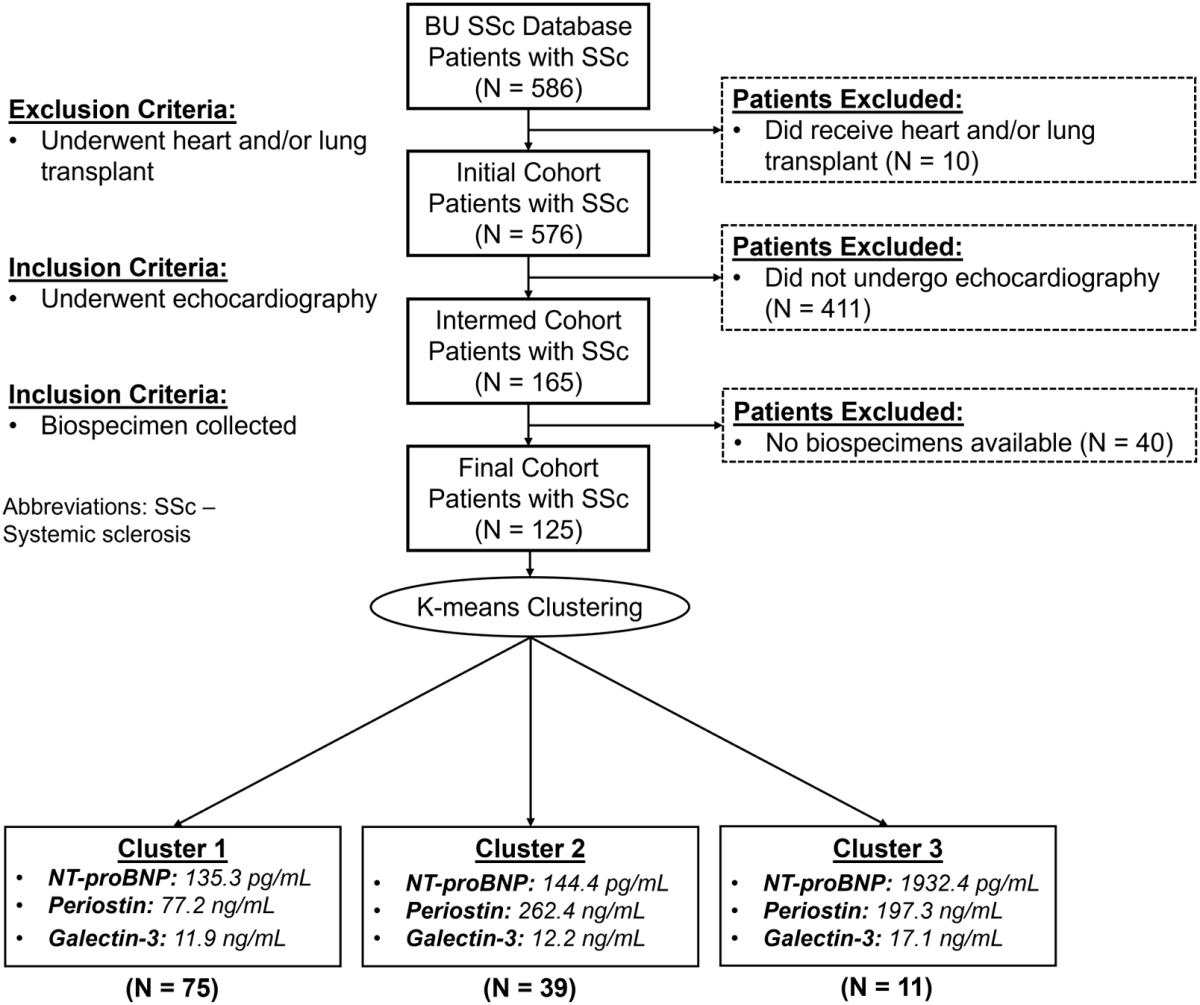
Study population. A K-means clustering was performed yielding 3 clusters based on serum biomarker levels of periostin, galectin-3, and N-terminal prohormone brain natriuretic peptide (NT-proBNP). The means of periostin, galectin-3, and NT-proBNP are presented in each cluster.

**Fig 2:**
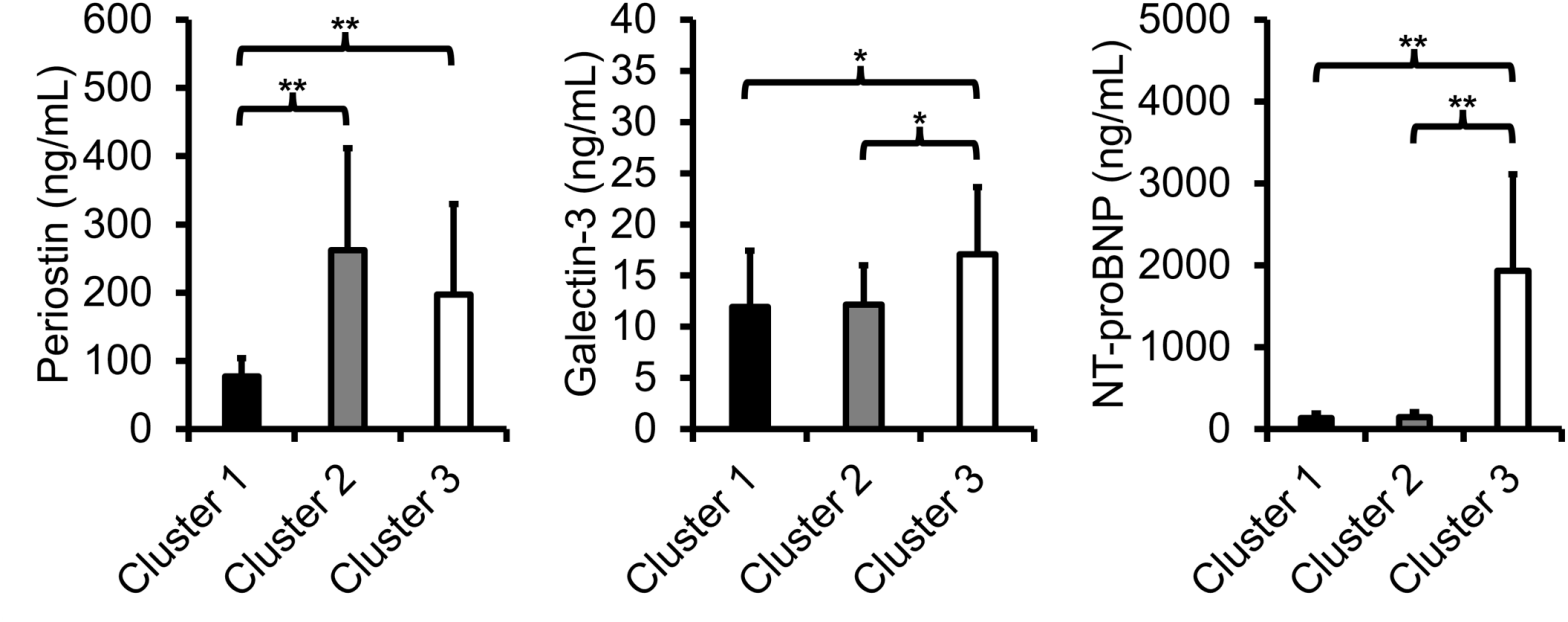
Serum biomarker levels of periostin, galectin-3, and N-terminal prohormone brain natriuretic peptide (NT-proBNP) within each cluster. The mean ± SD level of each serum biomarker is presented. *p < 0.05; **p < 0.001.

There were 165 patients (of the 586 with SSc) who had an echocardiogram performed at Boston Medical Center after excluding 10 who received a heart and/or lung transplant. We excluded 40 patients who did not have serum biospecimens; 125 patients comprised the final cohort (Fig 1). We grouped our serum biomarkers using K-means clustering to create 3 clusters: 1) *Cluster 1* (N = 75); 2) *Cluster 2* (N = 39); and 3) *Cluster 3* (N = 11). The biomarker levels of each cluster are shown in Fig 2. The median time between echocardiogram and biospecimen collection was 8.0 (IQR: 2.7, 20.8) months (See S4 Fig for a frequency distribution).

### Cluster description

#### Cluster 1.

Patients in *Cluster 1* exhibited lowest serum levels of all three biomarkers, with a mean periostin level similar to that we previously found in healthy controls [20]. Median disease duration was over 6 years, and patients had the lowest proportion of diffuse SSc. Comparison of disease characteristics between clusters revealed that *Cluster 1* patients had the mildest skin involvement, as evaluated on modified Rodnan skin score (mRSS) (Table 1). Although nearly half had SSc-ILD and a quarter had SSc-PH, lung disease was predominantly mild, with patients in this cluster having normal forced vital capacity (FVC) with moderate reductions in mean diffusing capacity for carbon monoxide (D_L_CO). On speckle tracking echocardiography, *Cluster 1* had a mean LV GLS within borderline normal range and displayed the greatest average RV free wall GLS of all three clusters. In summary, patients in *Cluster 1* had lowest biomarker levels, mildest skin and lung disease, and overall minimal LV and RV dysfunction by GLS.

**Table 1:** Demographics and clinical characteristics.

	Cluster 1 (N = 75)	Cluster 2 (N = 39)	Cluster 3 (N = 11)	*p*-value
**Demographics**				
Age, years	52.8 ± 12.9	49.8 ± 12.5	54.3 ± 13.8	0.419
Male sex	8 (10.7%)	6 (15.4%)	3 (27.3%)	0.235^α^
Current/Former tobacco use	39 (52.0%)	22 (56.4%)	3 (27.3%)	0.227
Diffuse SSc	16 (21.3%)	22 (56.4%)	5 (45.5%)	< 0.001^β^
SSc disease duration, months (median [IQR])	73.3 [42.2, 144.5]	29.8 [15.0, 67.0]	71.2 [24.7, 143.2]	< 0.001^β^
Modified Rodnan skin score (median [IQR])	4.0 [2.0, 8.0]	11.0 [2.0, 18.0]	8.0 [3.5, 29.5]	< 0.001^α^
SSc-PH	18 (24.0%)	12 (30.8%)	9 (81.8%)	< 0.001^α^
SSc-ILD	38 (50.7%)	24 (61.5%)	4 (36.4%)	0.283
**Overlapping Autoimmune Conditions**				
Sjogren’s syndrome	16 (21.3%)	8 (20.5%)	2 (18.2%)	1^α^
Systemic lupus erythematosus	3 (4.0%)	2 (5.1%)	3 (27.3%)	0.028^α^
Dermatomyositis/Polymyositis	2 (2.7%)	3 (7.7%)	0	0.461^α^
Rheumatoid arthritis	4 (5.3%)	0	0	0.516^α^
**Comorbid Conditions**				
Asthma/COPD	7 (9.3%)	5 (12.8%)	1 (9.1%)	0.818^α^
Obstructive sleep apnea	10 (13.3%)	3 (7.7%)	0	0.491^α^
Venous thromboembolism	6 (8.0%)	3 (7.7%)	4 (36.4%)	0.027^α^
Coronary artery disease	5 (6.7%)	1 (2.6%)	0	0.808^α^
Atrial fibrillation/Atrial flutter	6 (8.0%)	5 (12.8%)	5 (45.5%)	0.008^α^
Chronic Kidney Disease	2 (2.7%)	0	4 (36.4%)	< 0.001^α^
Diabetes mellitus	7 (9.3%)	2 (5.1%)	1 (9.1%)	0.690^α^
Hypertension	28 (37.3%)	11 (28.2%)	6 (54.5%)	0.245^α^
Hyperlipidemia	30 (40.0%)	12 (30.8%)	2 (18.2%)	0.315^α^
**Autoantibody Positivity**				
Antinuclear Antibody	58 (77.3%)	31 (79.5%)	6 (54.5%)	0.233^α^
Anti-topoisomerase I Antibody	36 (48.0%)	19 (48.7%)	1 (9.1%)	0.047^α^
**Pulmonary Function Testing**				
FEV_1_, % predicted	84.9 ± 17.8	78.3 ± 17.3	67.8 ± 20.4	0.002
FVC, % predicted	85.9 ± 20.1	78.5 ± 17.6	71.5 ± 14.2	0.006
FEV_1_/FVC, %	78.5 ± 6.9	79.6 ± 6.1	74.6 ± 10.3	0.417
D_L_CO, % predicted	62.0 ± 22.0	56.5 ± 20.2	55.1 ± 25.2	0.156
**Functional Capacity**				
NYHA Class III/IV	21 (28.0%)	12 (30.8%)	8 (72.7%)	0.019^α^
**Serum Biomarker Level**				
Periostin, ng/mL (median [IQR])	77.2 ± 27.1	262.4 ± 149.7	197.3 ± 132.6	< 0.001
Galectin-3, ng/mL (median [IQR])	11.9 ± 5.5	12.2 ± 3.8	17.1 ± 6.6	0.019
NT-proBNP, ng/mL (median [IQR])	135.3 ± 53.1	144.4 ± 61.6	1932.4 ± 1180.0	< 0.001
**Conventional Echocardiography**				
Aortic regurgitation	6 (8.0%)	3 (7.7%)	2 (18.2%)	0.427^α^
Mitral regurgitation	17 (22.7%)	5 (12.8%)	4 (36.4%)	0.212^α^
Tricuspid regurgitation	26 (34.7%)	21 (53.8%)	8 (72.7%)	0.019^α^
Pulmonic regurgitation	13 (17.3%)	7 (17.9%)	2 (18.2%)	1^α^
LA diameter, mm	32.8 ± 5.1	33.0 ± 5.9	34.6 ± 8.6	0.403
LA volume index, mL/m^2^	23.2 ± 8.6	23.9 ± 6.9	24.4 ± 11.3	0.600
LA dilation	8 (10.7%)	3 (7.7%)	3 (27.3%)	0.168^α^
IVSd, mm	8.6 ± 1.8	8.6 ± 2.0	9.0 ± 2.1	0.719
LVEDd diameter, mm	43.3 ± 7.3	45.2 ± 4.8	42.2 ± 8.2	0.721
PWd, mm	8.5 ± 1.7	8.7 ± 1.6	8.9 ± 2.1	0.322
LVESd, mm	28.7 ± 4.8	29.6 ± 4.7	29.1 ± 6.7	0.472
LV mass index, g/m^2^	67.7 ± 18.0	72.6 ± 20.9	68.7 ± 35.8	0.455
LV ejection fraction, %	62.7 ± 5.7	62.4 ± 8.6	61.8 ± 6.4	0.655
Mitral E wave velocity, cm/s	80.7 ± 18.9	79.5 ± 19.7	78.7 ± 22.6	0.697
Mitral A wave velocity, cm/s	75.2 ± 24.1	65.5 ± 24.2	67.2 ± 17.8	0.062
Mitral E and A velocity ratio	1.2 ± 0.4	1.3 ± 0.4	1.2 ± 0.5	0.144
Septal e’ velocity, cm/s	8.0 ± 2.6	8.4 ± 2.5	7.5 ± 3.1	0.962
Lateral e’ velocity, cm/s	10.5 ± 3.8	10.9 ± 3.5	10.1 ± 3.3	0.979
Mitral E and e’ ratio	14.1 ± 6.9	12.6 ± 3.5	13.1 ± 2.3	0.258
Mitral E and e’ ratio > 15	25 (33.3%)	9 (23.1%)	3 (27.3%)	0.571^α^
TRV > 2.8 m/s	39 (52.0%)	25 (64.1%)	10 (90.9%)	0.033^α^
LV diastolic dysfunction	20 (26.7%)	7 (17.9%)	2 (18.2%)	0.160^α^
LV wall thickening	11 (14.7%)	9 (23.1%)	1 (9.1%)	0.465^α^
RA dilation	9 (12.0%)	5 (12.8%)	7 (63.6%)	< 0.001^α^
RV dilation	9 (12.0%)	3 (7.7%)	8 (72.7%)	< 0.001^α^
RV systolic dysfunction	3 (4.0%)	3 (7.7%)	6 (54.5%)	< 0.001^α^
Pericardial effusion	13 (17.3%)	5 (12.8%)	6 (54.5%)	0.012^α^
**Speckle-Tracking Echocardiography**				
LV GLS, %	17.7 ± 2.6	17.7 ± 3.0	14.8 ± 2.6	0.015
RV Free Wall GLS, %	23.3 ± 5.7	20.9 ± 5.5	14.9 ± 5.0	< 0.001

**Abbreviations: COPD** – Chronic obstructive pulmonary disease; **D**_**L**_**CO** – Diffusing capacity for carbon monoxide; **FEV**_**1**_ – Forced expiratory volume in 1 second; **FVC** – Forced vital capacity; **GLS –** Global longitudinal strain; **IVSd** – Interventricular septal diameter; **LA** – Left atrial; **LV** – Left ventricular; **LVEDd** – Left ventricular end-diastolic diameter; **LVESd** – Left ventricular end-systolic diameter; **NT-proBNP** – N-terminal prohormone brain natriuretic peptide; **NYHA** – New York Heart Association; **PWd** – Posterior wall diameter; **RA** – Right atrial; **RV** – Right ventricular; **SSc** – Systemic sclerosis; **SSc-ILD** – Systemic sclerosis-related interstitial lung disease; **SSc-PH** – Systemic sclerosis-related pulmonary hypertension; **TRV** – Tricuspid regurgitant velocity.

Data are presented in mean ± SD for continuous variables and n (%) for categorical variables unless otherwise specified. The *p*-values were obtained using unpaired t-test for continuous variables and chi-squared test for categorical variables unless otherwise stated (^α^Fisher Test; ^β^Wilcoxon Test).

#### Cluster 2.

Patients in *Cluster 2* exhibited elevated periostin levels with similar NT-proBNP and galectin-3 levels as *Cluster 1*. Compared to the other two clusters, the median SSc disease duration was shortest in *Cluster 2* (< 3 years), although these patients had the greatest severity of skin disease by mRSS (Table 1). More than half of these patients had diffuse SSc. A greater percentage of patients with SSc-ILD was observed in *Cluster 2* compared to the other two clusters. Patients in *Cluster 2* exhibited predominantly mild ILD by FVC. Although exhibiting a similar mean LV GLS compared to those in Cluster 1, patients in Cluster 2 also had a greater LV mass index and proportion of patients with LV wall thickening. Furthermore, while the percentage of patients with SSc-PH were similar in both Cluster 1 and Cluster 2, patients in Cluster 2 had a greater degree of RV systolic dysfunction reflected by lower RV free wall GLS values and greater tricuspid regurgitant velocities. To conclude, patients in *Cluster 2* showed isolated elevations in periostin levels, early disease duration, a higher skin score, and intermediate echocardiographic assessments suggestive of possible PH.

#### Cluster 3.

Despite a similar median SSc disease duration to those in *Cluster 1*, patients in *Cluster 3* had elevations in all three biomarkers, and a higher mRSS skin score (Table 1). Among all three clusters, the greatest percentage of patients with SSc-PH was observed in *Cluster 3* with nearly three-quarters exhibiting severe functional limitation (New York Heart Association class III/IV). On echocardiography, there was a greater percentage of right-sided structural abnormalities in *Cluster 3* compared to the other two clusters. This cluster exhibited the greatest percentage of patients with abnormal LA volume index. These patients also had the greatest reductions in both LV and RV free wall GLS. In brief, *Cluster 3* patients had elevations in all three biomarkers and the most pronounced cardiovascular abnormalities.

### Regional distribution of longitudinal strain

**Fig 3:**
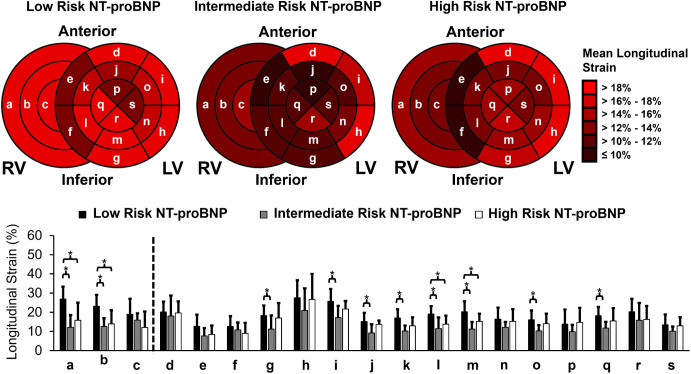
Left ventricular (LV) and right ventricular (RV) free wall longitudinal strain distribution in low risk, intermediate risk, and high risk NT-proBNP groups. Bull’s eye plot (top panel) and histogram (bottom panel) of longitudinal strain at the following cardiac segments: a) RV basal; b) RV mid; c) RV apical; d) LV basal anterior; e) LV basal anteroseptal; f) LV basal inferoseptal; g) LV basal inferior; h) LV basal inferolateral; i) LV basal anterolateral; j) LV mid anterior; k) LV mid anteroseptal; l) LV mid inferoseptal; m) LV mid inferior; n) LV mid inferolateral; o) LV mid anterolateral; p) LV apical anterior; q) LV apical septal; r) LV apical inferior; s) LV apical lateral. LV GLS for the low risk, intermediate risk, and high risk NT-proBNP groups were 17.9 ± 2.5%, 12.4 ± 2.9%, and 15.4 ± 2.4%, respectively. RV free wall GLS for the low risk, intermediate risk, and high risk NT-proBNP groups were 22.8 ± 5.5%, 13.7 ± 3.9%, and 14.3 ± 6.0%, respectively. *p < 0.05.

For risk stratification by NT-proBNP, alone, patients with SSc were divided into low (< 300 pg/mL; N = 111), intermediate (≥ 300 pg/mL and < 1,100 pg/mL; N = 7), and high (≥ 1,100 pg/mL; N = 7) risk groups [35]. From the regional distribution of longitudinal strain by NT-proBNP (Fig 3), there were 8 (42.1%) segments exhibiting a mean longitudinal strain < 18%, indicative of depressed myocardial deformation, in the low risk NT-proBNP group compared to 17 (89.5%) segments in the intermediate risk NT-proBNP group and 16 (84.2%) segments in the high risk NT-proBNP group. LV and RV free wall GLS were significantly reduced in both intermediate and high risk NT-proBNP groups compared to the low risk NT-proBNP group. Over all three risk groups, the greatest reduction in LV longitudinal strain was observed at the basal septal segments. With respect to the RV free wall, there were reductions in longitudinal strain across all segments in both the intermediate and high risk NT-proBNP groups. In summary, even in the low risk NT-proBNP group, nearly half of the myocardial segments exhibited abnormal strain patterns, indicating subclinical cardiac involvement in patients with SSc. Notably, there was not a significant difference in longitudinal strain observed in each individual segment between intermediate and high NT-proBNP groups.

**Fig 4:**
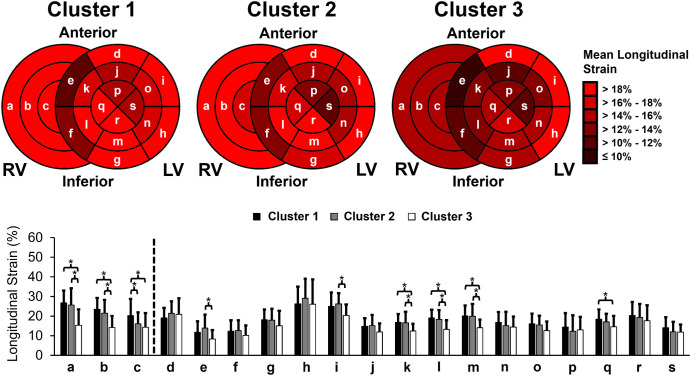
Left ventricular (LV) and right ventricular (RV) free wall longitudinal strain distribution in each cluster. Bull’s eye plot (top panel) and histogram (bottom panel) of longitudinal strain at the following cardiac segments: a) RV basal; b) RV mid; c) RV apical; d) LV basal anterior; e) LV basal anteroseptal; f) LV basal inferoseptal; g) LV basal inferior; h) LV basal inferolateral; i) LV basal anterolateral; j) LV mid anterior; k) LV mid anteroseptal; l) LV mid inferoseptal; m) LV mid inferior; n) LV mid inferolateral; o) LV mid anterolateral; p) LV apical anterior; q) LV apical septal; r) LV apical inferior; s) LV apical lateral. LV GLS for Cluster 1, Cluster 2, and Cluster 3 were 17.7 ± 2.6%, 17.7 ± 3.0%, and 14.8 ± 2.6%, respectively. RV free wall GLS for Cluster 1, Cluster 2, and Cluster 3 were 23.3 ± 5.7%, 20.9 ± 5.5%, and 14.9 ± 5.0%, respectively. *p < 0.05.

In contrast, from the regional distribution of longitudinal strain by cluster (Fig 4), there were 16 (84.2%) segments with average longitudinal strain < 18% in *Cluster 3*, compared to 11 (57.9%) in *Cluster* 2 and 8 (42.1%) in *Cluster 1*. With respect to the RV free wall, there were reductions in longitudinal strain across all segments in *Cluster 3* compared to only the apical RV free wall in *Cluster 2*. The greatest reductions in LV longitudinal strain were seen at the basal septal segments in all clusters. Overall, when patients were stratified using all three biomarkers, the number of affected segments and the severity of strain reduction appeared to increase with progression from *Cluster 1* to *Cluster 2* and from *Cluster 2* to *Cluster 3*. Overall, there was a clearer distinction between each group using NT-proBNP, periostin, and galectin-3 compared to NT-proBNP, alone.

### Detection of cardiac involvement by multi-biomarker clustering

The performance characteristics of the multi-biomarker clustering are summarized in Table 2. Whether using NT-proBNP, alone, or a multi-biomarker clustering we detected RV dysfunction with a greater sensitivity, specificity, and accuracy compared to LV dysfunction by GLS. However, compared to risk grouping by NT-proBNP, alone, our multi-biomarker clustering achieved a greater sensitivity although at the cost of specificity in detecting abnormal LV and RV function by GLS. However, the overall accuracy using our multi-biomarker clustering approach was similar to using NT-proBNP, alone.

**Table 2:** Performance characteristics of multi-biomarker clustering.

Algorithm	Sensitivity	Specificity	Accuracy
*Abnormal Left Ventricular Function*			
Intermediate/High Risk NT-proBNP	0.19 (0.10, 0.30)	0.98 (0.90, 1.00)	0.54 (0.45, 0.63)
Multi-Biomarker Cluster 2/3	0.45 (0.33, 0.57)	0.66 (0.52, 0.78)	0.54 (0.45, 0.63)
*Abnormal Right Ventricular Function*			
Intermediate/High Risk NT-proBNP	0.24 (0.13, 0.38)	0.99 (0.92, 1.00)	0.66 (0.57, 0.75)
Multi-Biomarker Cluster 2/3	0.59 (0.45, 0.72)	0.75 (0.63, 0.84)	0.68 (0.59, 0.76)

**Definitions: Abnormal left ventricular function** – Left ventricular global longitudinal strain < 18%; **Abnormal right ventricular function** – Right ventricular free wall global longitudinal strain < 20%; For each performance characteristic, 95% confidence intervals are shown.

Given that periostin may be elevated in skin sclerosis [20,21], a secondary analysis was also performed comparing periostin levels in patients with and without LV dysfunction by GLS among those with diffuse and non-diffuse SSc (i.e., limited or sine) (S5 Fig). In patients with diffuse SSc (N = 40), there was no statistically significant difference in periostin levels between those with (N = 17) and without LV dysfunction (N = 23). However, following logarithmic transformation, there was a trend toward more elevated periostin levels in patients with LV dysfunction compared to those without (7.5 ± 1.1 ng/mL vs. 6.9 ± 0.9 ng/mL, p = 0.053) among those with diffuse SSc. In patients with non-diffuse SSc (N = 53), there was no statistically significant difference in periostin levels between those with (N = 20) and without LV dysfunction (N = 33), even following logarithmic transformation.

### Association with all-cause mortality

**Fig 5:**
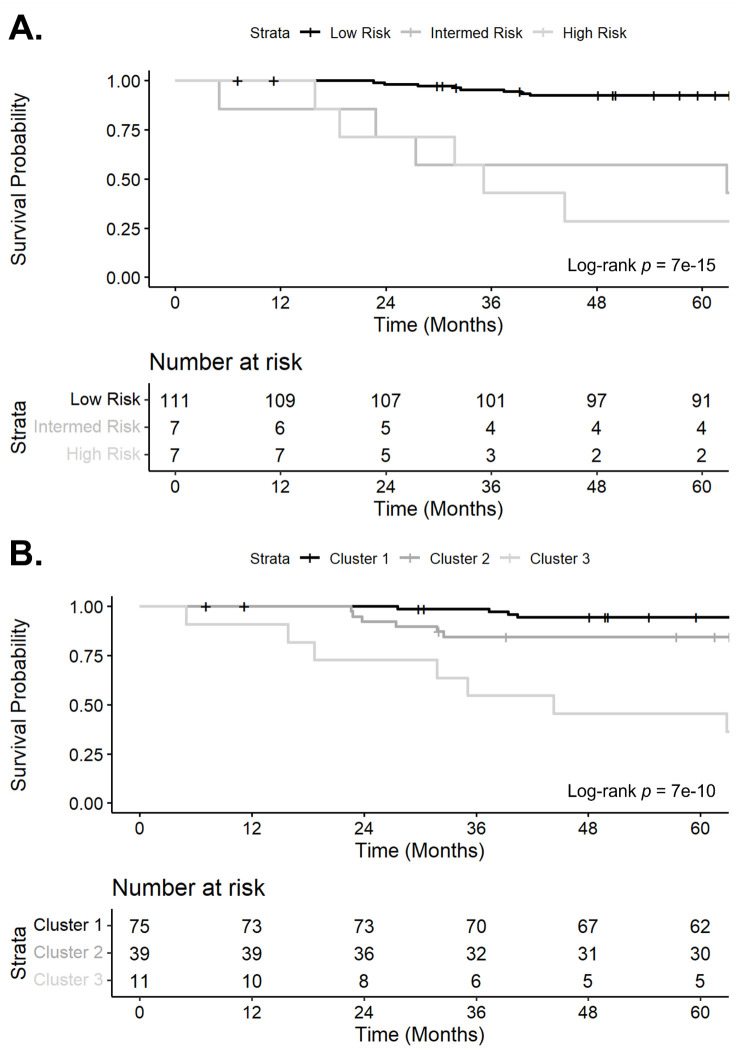
Kaplan-Meier survival curve by A.) NT-proBNP risk groups and B.) Cluster. Vertical bars denote censored data.

In the cohort of 125 patients, there were 25 (20.0%) deaths. After excluding the 18 (14.4%) who were lost to follow-up, the 1-year and 3-year all-cause mortality rates were 0.9% and 11.2%, respectively. Of the 12 patients who died within 3 years, 3 (25.0%) were in the intermediate risk NT-proBNP group, and 4 (33.3%) were in the high risk NT-proBNP group. On the Kaplan-Meier survival curve (Fig 5A), there was a statistically significant difference between NT-proBNP risk groups. Median survival for the intermediate and high risk NT-proBNP groups were 62.8 months and 35.1 months, respectively. When adjusted by age, male sex, SSc disease duration, and FVC, patients in the intermediate risk NT-proBNP group had a HR of 9.35 (95% CI: 3.12, 28.05) for all-cause mortality compared to those in the low risk NT-proBNP group. In contrast, patients in the high risk NT-proBNP group had an adjusted HR of 13.80 (95% CI: 4.79, 39.78) for all-cause mortality compared to those in the low risk NT-proBNP group. Sensitivity analyses arrived at similar findings.

Of the same 12 patients who died within 3 years, 6 (50.0%) were classified in *Cluster 2*, and 5 (41.7%) were classified in *Cluster 3*. On the Kaplan-Meier survival curve (Fig 5B), there was a statistically significant difference between clusters. Median survival for *Cluster 3* was 44.3 months. When adjusted by age, male sex, SSc disease duration, and FVC, patients in *Cluster 2* and *Cluster 3* had a HR of 2.72 (95% CI: 0.92, 8.02) and 14.42 (95% CI: 4.82, 43.18) respectively for all-cause mortality compared to those in *Cluster 1*. When we performed our two sensitivity analyses: the first to exclude patients who were lost to follow-up; and the second to exclude patients who had a greater than a 1-year time window between echocardiography and serum collection, we arrived at similar findings.

## Discussion

SSc is a complex disease with multi-organ involvement, where PH significantly contributes to mortality. Despite being classified as Group 1 pulmonary arterial hypertension (PAH) [35], outcomes in SSc-PH are worse than in idiopathic PAH [36], potentially due to concurrent left-sided cardiac disease [1–3]. Identifying early biomarkers of SSc cardiac involvement is crucial for patient risk stratification, understanding disease mechanisms, and identifying novel therapeutic targets. In this study, we measured NT-proBNP, a widely utilized and well-validated biomarker of congestive heart failure, and two novel protein markers of fibrosis recently found to be associated with SSc, periostin and galectin-3 [20,24]. Here, we present evidence that using a multi-biomarker approach may offer superior insights into cardiac involvement and mortality risk compared to relying solely on NT-proBNP.

NT-proBNP has been an often used biomarker of cardiovascular diseases [18]. Our study revealed that when stratified based on NT-proBNP expression alone, nearly half of the myocardial segments exhibited abnormal strain patterns in the low NT-proBNP group, indicating some degree of subclinical cardiac dysfunction. Notably, there was not a statistically significant difference in longitudinal strain on a segmental level between the intermediate and high NT-proBNP groups. These findings suggest that abnormalities in myocardial deformation may be present even at relatively low NT-proBNP levels in patients with SSc, and that the relationship between NT-proBNP and longitudinal strain may not necessarily be linear in this patient population. Furthermore, given that only 58.3% of the patients who died within 3 years were in either intermediate or high risk NT-proBNP risk groups, NT-proBNP on its own might not be an effective predictor of mortality in SSc. It is also worth noting that NT-proBNP may be abnormally elevated by impaired renal function such as in chronic kidney disease that can occur in patients with SSc renal involvement [37–39]. Finally, in our study NT-proBNP, by itself, was not a sensitive marker of abnormal cardiac function. In most clinical settings, NT-proBNP is often used in conjunction with other testing to create a profile for mortality risk [35].

To complement our non-invasive diagnostic approach, we incorporated periostin and galectin-3 as surrogate indicators of cardiac fibrosis in SSc [20,24]. By utilizing all three biomarkers, we clustered patients into three distinct groups, each exhibiting different alterations in longitudinal strain on echocardiography and varying all-cause mortality. Over all 3 clusters, the greatest decline in longitudinal strain was localized at the basal segments of the interventricular septum which appeared to be associated with increasing degrees of RV dysfunction, consistent with prior findings [2,40]. Changes in the basal septum have been observed in the early stages of LV remodeling. With increasing severity of PH, these changes are likely accelerated with RV pressure overload which impacts the structure of the interventricular septum, ultimately affecting LV contractility [6,41,42].

*Cluster 1*, characterized by “normal-like” biomarker expression, showed the mildest degree of cardiac dysfunction by longitudinal strain and the best survival outcomes. *Cluster 2* displayed isolated periostin elevation with a shorter disease duration, the most advanced skin disease, and mild changes in longitudinal strain on a segmental level. Lastly, *Cluster 3* exhibited elevations in all three biomarkers, the greater reduction in both regional and global longitudinal strain, and the highest all-cause mortality. Moreover, *Cluster 3* appears to have characteristics of both left and right cardiac abnormalities. It may be important with larger cohorts to further classify contributions from primary heart involvement and PH. By incorporating periostin and galectin-3 to NT-proBNP, we were able to increase sensitivity for the detection of abnormalities in cardiac function by LV and RV free wall GLS.

The observation that patients with isolated elevation in periostin expression exhibit an intermediate cardiac phenotype and the earliest disease duration suggests that periostin may be an early indicator of cardiac involvement. This is further corroborated by our previous findings that periostin is absent in cardiac tissue in control subjects but upregulated in SSc, even in areas without established collagen deposition [20]. These insights imply that periostin may be involved in the initial stages of cardiac remodeling, potentially preceding structural changes typically linked to more advanced disease. While patients with elevated circulating levels may be affected by skin fibrosis, patients with greater severity of skin disease have also been previously found to have more severe cardiac involvement in SSc [43,44]. In a subset analysis, we found that there was trend toward elevated log-transformed periostin levels in patients with LV dysfunction compared to those without among patients with diffuse SSc, which may have been limited by the small sample size. Interestingly, serum periostin has also been found to be increased in patients following myocardial infarction, and it predicted adverse cardiac remodeling months later [45]. Unlike patients with SSc, these patients had no skin disease.

In the current study, elevations in galectin-3 were seen together with those in NT-proBNP in *Cluster 3* and associated with reduced longitudinal strain both regionally and globally, and with the highest all-cause mortality. These results suggest that galectin-3 may be a late marker of fibrosis. This was consistent with previous reports of elevated circulating galectin-3 in mid-to-late stage disease, and its association with LV dysfunction on echocardiography in SSc [22,24].

### Study limitations

There were limitations to our study. The retrospective nature of our observational data resulted in inconsistent timing of assessments and missing data, which we addressed using multiple imputation by chained equations. However, not all variables were missing completely at random. There were 3 variables that had > 15% missing: 1) Anti-topoisomerase antibody; 2) Antinuclear antibody; and 3) Tricuspid regurgitant velocity. For these variables, we ran a logistic regression on these variables using: a) Age; b) Sex; c) Diffuse SSc; d) SSc disease duration; and e) PH. None of the variables showed an association with missing values for antinuclear antibody. However, missing values for anti-topoisomerase antibody was associated with diffuse SSc, which was expected given that positive anti-topoisomerase antibody is a marker of diffuse SSc. Missing values for tricuspid regurgitant velocity was associated with PH, which was also expected given that PH often results in a measurable tricuspid regurgitant velocity. Furthermore, 36% of patients with PH may be absent of a measurable tricuspid regurgitant velocity [46].

While incorporating periostin and galectin-3 to NT-proBNP improved our sensitivity for the detection of abnormal cardiac function using GLS compared to NT-proBNP, alone, our approach can be better improved by incorporating additional biomarkers. Regarding whether periostin may represent an early marker of cardiac involvement in SSc, future longitudinal analysis will be necessary to determine whether elevated serum periostin levels precede or follow fibrosis and cardiac disease. Likewise, because the study design precluded tracking longitudinal changes in biomarker levels, the duration of time between echocardiography and blood sample may be important. The median time between echocardiogram and biospecimen collection was 8 months during which there may be progression of disease leading to alterations in protein expression. However, we arrived at similar findings by sensitivity analysis when restricting to only patients who had both echocardiogram and biospecimen collection within 1 year.

Other potential limitations are related to image quality of the echocardiograms for speckle-tracking analysis, and a small sample size. Consequently, our findings should be considered hypothesis-generating, requiring validation through larger, prospective, multicenter studies.

## Conclusion

In conclusion, combining NT-proBNP, periostin, and galectin-3 as serum biomarkers enhances risk stratification and early detection of cardiac involvement in patients with SSc. Our findings show that incorporating a multi-biomarker approach may help improve management of cardiac complications in systemic sclerosis and potentially enable more personalized strategies to improve patient outcomes. However, before implementation in routine care, further prospective studies must refine biomarker sensitivity, specificity, and accuracy together with optimizing detection strategies and establishing clinical protocols for integration.

## Supporting information

S1 Fig Histogram of biomarkers before (left panel) and after logarithmic transformation by base 2 (right panel). (TIF)

S2 Fig Optimal number clusters determined by A.) Elbow method and B.) Calinski-Harabasz Index. The optimal cluster was 3. (TIF)

S3 Fig Cluster plot of the principal components. The two components explain 75.7% of the point variability.
(TIF)

S4 Fig Frequency distribution of time in months between serum biospecimen collection and echocardiogram. The median (IQR) time between serum biospecimen collection and echocardiogram was 8.0 (IQR: 2.7, 20.8) months.
(TIF)

S5 Fig Comparison of periostin levels in patients with and without LV dysfunction. A.) Periostin levels and B.) Log-transformed periostin levels in patients with diffuse SSc. C.) Periostin levels and D.) Log-transformed periostin levels in patients with limited/sine SSc.
(TIF)

S1 Table Summary of missing data. Abbreviations: FEV_1_ - forced expiratory volume in 1 second; FVC - forced vital capacity. (XLSX)
