# Supplementary figures and images for: A multi-biomarker approach to risk stratification and detection of early cardiac disease in systemic sclerosis
Source: PLoS One. 2025 Jul 31;20(7):e0328734. doi: 10.1371/journal.pone.0328734 (PMC12312888; doi:10.1371/journal.pone.0328734)

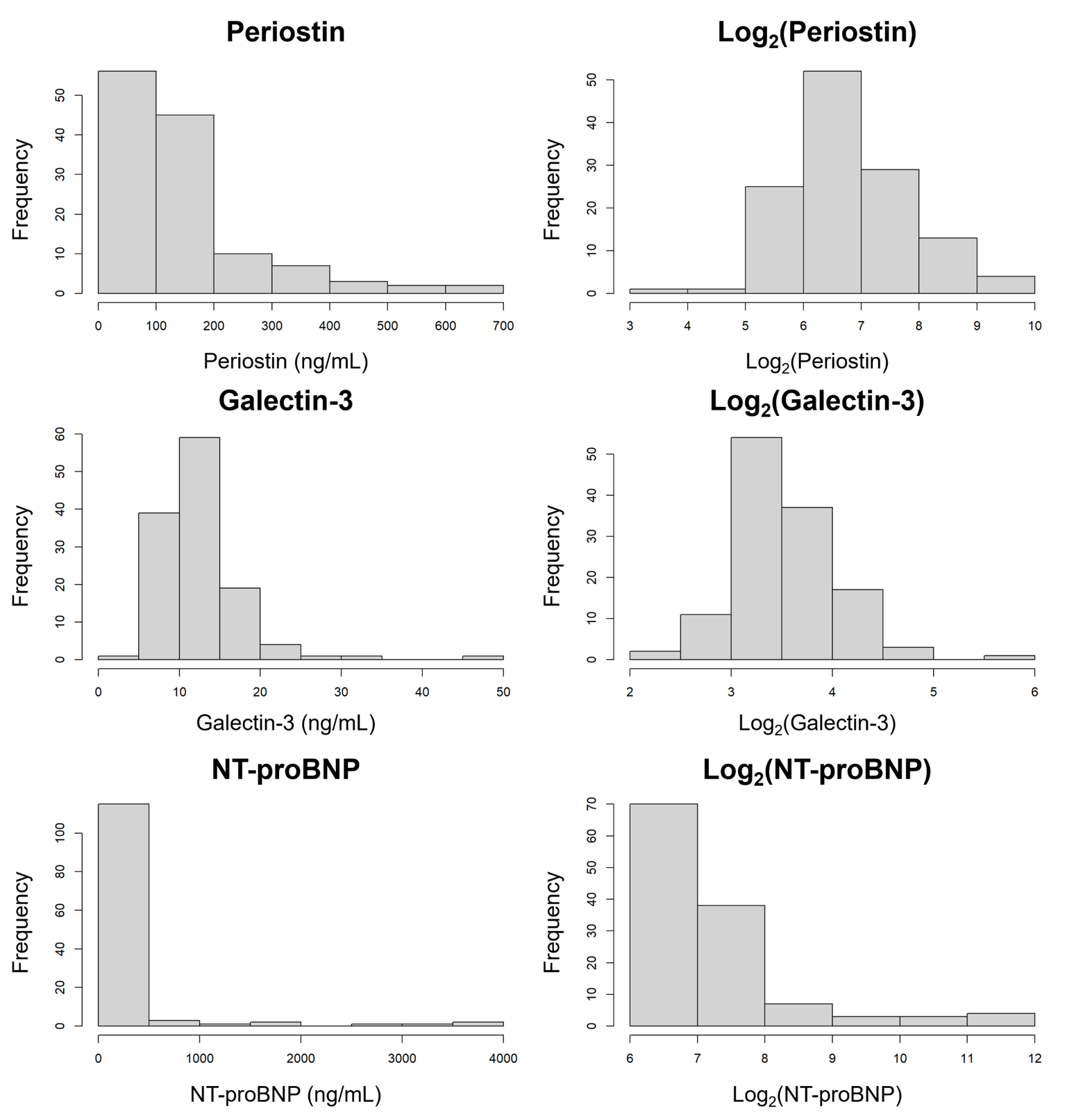

Supplement: S1 Fig — (TIF) [file pone.0328734.s001.tif]

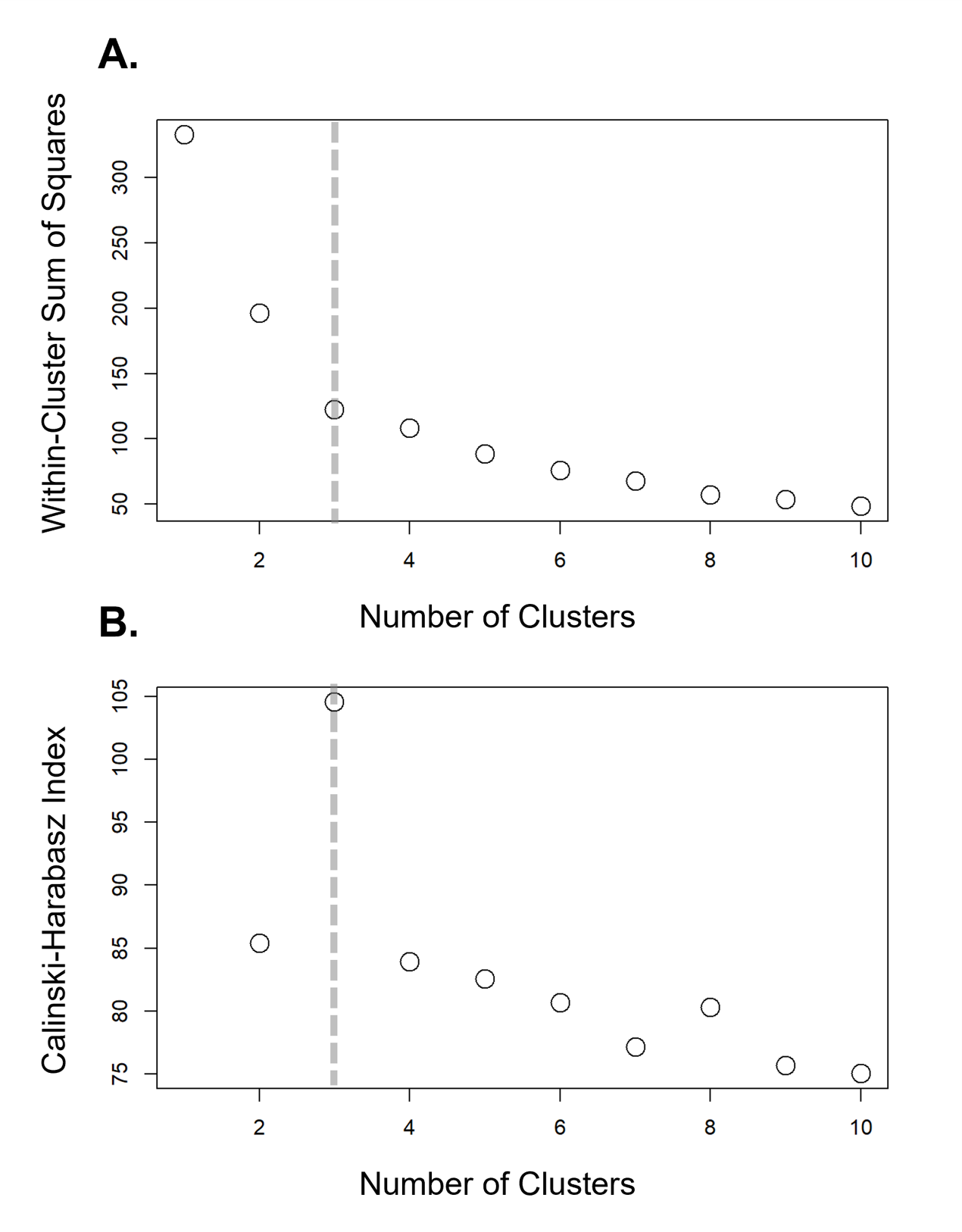

Supplement: S2 Fig — (TIF) [file pone.0328734.s002.tif]

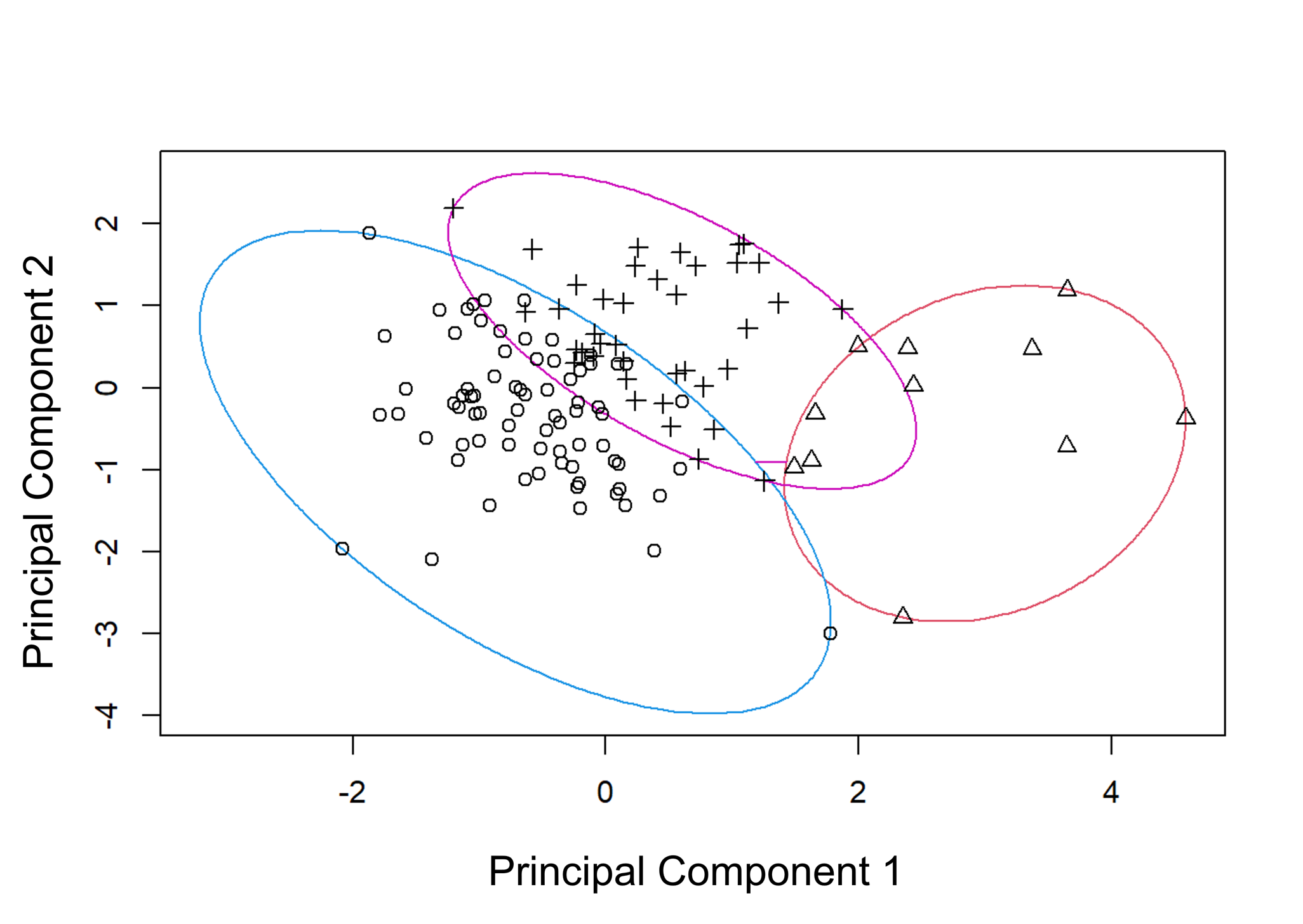

Supplement: S3 Fig — The two components explain 75.7% of the point variability. (TIF) [file pone.0328734.s003.tif]

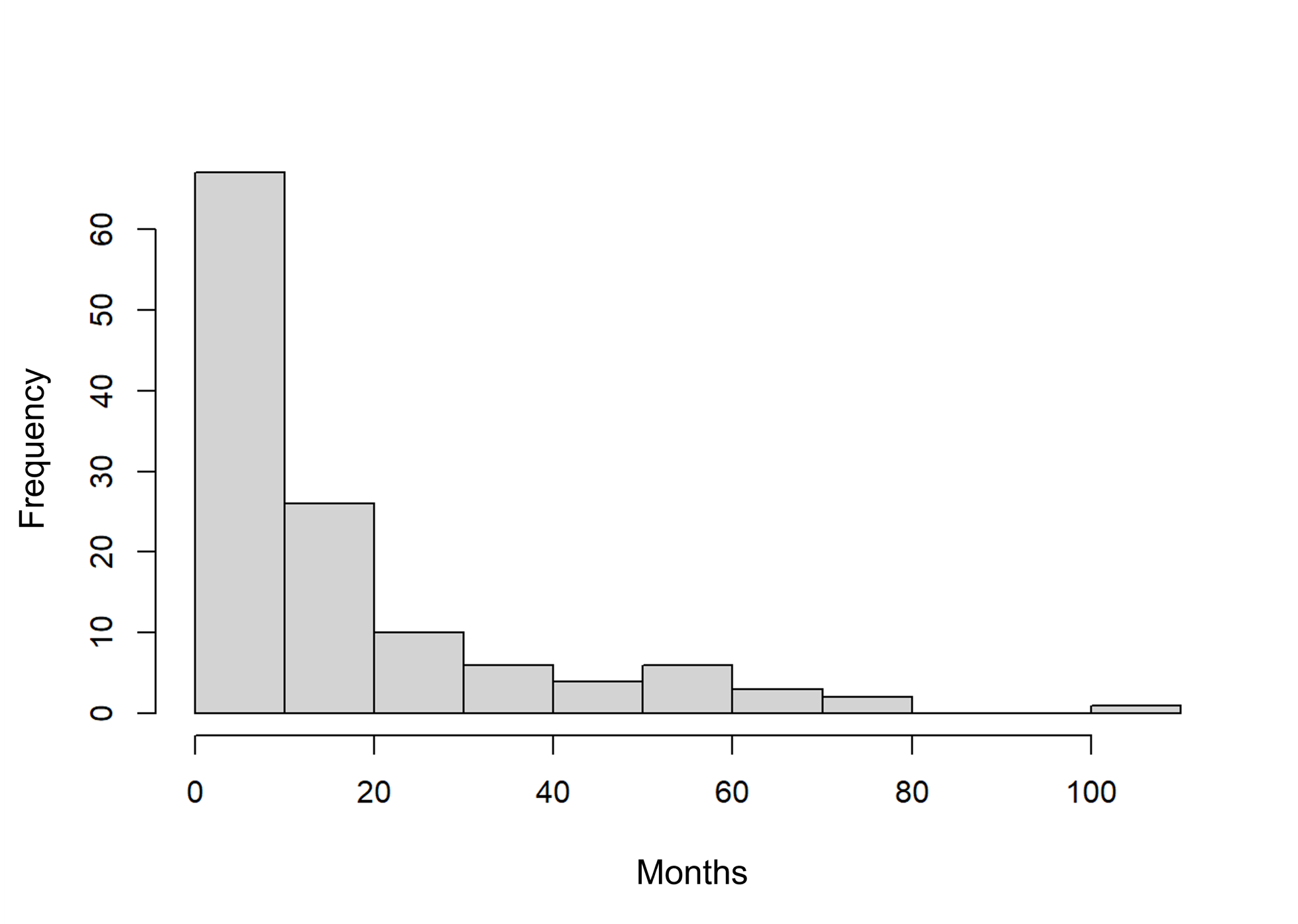

Supplement: S4 Fig — The median (IQR) time between serum biospecimen collection and echocardiogram was 8.0 (IQR: 2.7, 20.8) months. (TIF) [file pone.0328734.s004.tif]

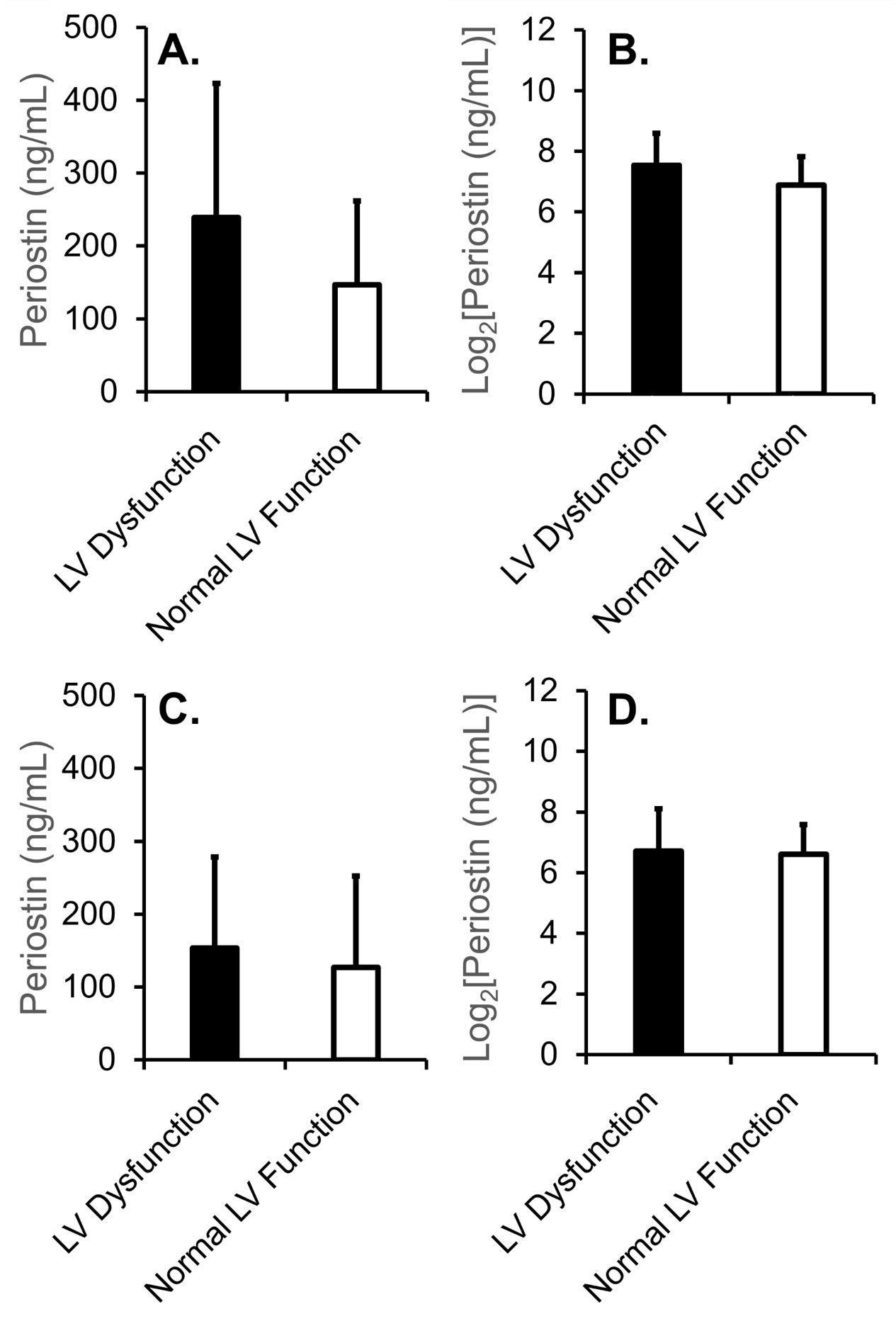

Supplement: S5 Fig — A.) Periostin levels and B.) Log-transformed periostin levels in patients with diffuse SSc. C.) Periostin levels and D.) Log-transformed periostin levels in patients with limited/sine SSc. (TIF) [file pone.0328734.s005.tif]
